# Preparation of Silk Fibroin/Carboxymethyl Chitosan Hydrogel under Low Voltage as a Wound Dressing

**DOI:** 10.3390/ijms22147610

**Published:** 2021-07-16

**Authors:** Zhenyu Chen, Xiaoning Zhang, Jianwei Liang, Yansong Ji, Yuqing Zhou, Hao Fang

**Affiliations:** State Key Laboratory of Silkworm Genome Biology, College of Sericulture, Textile and Biomass Sciences, Southwest University, Chongqing 400715, China; c618@email.swu.edu.cn (Z.C.); liangjw@email.swu.edu.cn (J.L.); jys2020@email.swu.edu.cn (Y.J.); zhouxguai@163.com (Y.Z.); fangritian@email.swu.edu.cn (H.F.)

**Keywords:** silk fibroin, carboxymethyl chitosan, electrodeposition, wound dressing

## Abstract

At present, silk fibroin (SF) hydrogel can be prepared by means of electrodeposition at 25 V in direct current (DC) mode. Reducing the applied voltage would provide benefits, including lower fabrication costs, less risk of high voltage shocks, and better stability of devices. Here, a simple but uncommon strategy for SF-based hydrogel preparation using 4 V in DC mode is discussed. SF was mixed and cross-linked with carboxymethyl chitosan (CMCS) through hydrogen bonding, then co-deposited on the graphite electrode. The thickness, mass, and shape of the SF/CMCS hydrogel were easily controlled by adjusting the electrodeposition parameters. Morphological characterization of the prepared hydrogel via SEM revealed a porous network within the fabricated hydrogel. This structure was due to intermolecular hydrogen bonding between SF and CMCS, according to the results of thermogravimetric analysis and rheological measurements. As a potential wound dressing, SF/CMCS hydrogel maintained a suitable moisture environment for wound healing and demonstrated distinct properties in terms of promoting the proliferation of HEK-293 cells and antibacterial activity against *Escherichia coli* and *Staphylococcus aureus*. Furthermore, histological studies were conducted on a full-thickness skin wound in rats covered with the SF/CMCS hydrogel, with results indicating that this hydrogel can promote wound re-epithelization and enhance granulation tissue formation. These results illustrate the feasibility of using the developed strategy for SF-based hydrogel fabrication in practice for wound dressing.

## 1. Introduction

Silk fibroin (SF) is the major protein in the silk produced by *Bombyx mori*. After degumming, silk fibroin fiber can be dissolved in a highly concentrated salt solution [1]. This solution is called regenerated silk fibroin (RSF) solution once it is desalinated. RSF is characterized by a slow biodegradation rate, suitable cellular response, and high biocompatibility [2]. In addition, RSF can be reconstituted in various forms [3,4,5,6]. Among these forms, SF-based hydrogel is particularly attractive as it can provide a three-dimensional structure, has a high water content, soft consistency, and excellent water stability, and can closely adhere to the skin [7]. SF-based hydrogel can be prepared through the addition of a chemical cross-linking agent [8], sonication [9], or application of an electric current [10]. The use of a chemical cross-linking agent reduces the biocompatibility of the hydrogel [8], whereas sonication-induced gelation suffers from slow gel formation and an unstable structure [11]. SF-based hydrogel prepared via electrogelation is free from chemical cross-linking agents and can be achieved over a short and controllable period [12]. At present, SF electrogelation can be obtained within 5 min under a 25 V direct current (DC) voltage [10,13]. Reducing the driving voltage of SF electrogelation can save energy, avoid potential electrochemical reactions when working with high voltages, and lower the fabrication cost for an electrogelation device, which should decrease the fabrication cost and increase product stability.

Carboxymethyl chitosan (CMCS) is a chitosan (the second most abundant polysaccharide in nature [14]) derivative with properties including biocompatibility, antibacterial activity, and moisture retention. In addition, it can participate in free radical scavenging as an antioxidant [14]. Wang et al. fabricated a carboxylated chitosan-derived film based on coordinated electrodeposition [15], in which the silver electrode underwent anodic electrochemical oxidation and then generated Ag^+^ ions in situ. These in situ-generated Ag^+^ ions can coordinate with the carboxylated chitosan adjacent to the silver electrode (carboxylated chitosan has abundant -COOH, -NH_2_, and -OH groups) and generate a composite film that can be peeled from the silver electrode [16]. Because the Ag^+^ ions are released near the electrode surface, the thickness of the fabricated composite film was limited within the micrometer scale. In addition, carboxylated chitosan-derived film was prepared with the aid of metal ions, which are potentially biotoxic.

It was reported that chitosan can self-associate to form a three-dimensional hydrogel network via a cathodic neutralization mechanism [17]. The chitosan molecules carry positive charges through the protonation of amine groups in an acidic aqueous environment and are attracted to the cathode surface by electrostatic attraction. Subsequently, positively charged chitosan molecules are neutralized by hydroxide ions generated on the cathode surface, which are then deposited and undergo self-association to form the hydrogel [18,19]. Upon deprotonation of the carboxyl groups, CMCS would carry a negative charge in an alkaline solution [20]. We therefore believe that the fabrication of CMCS hydrogel, without the involvement of metal ions, by means of electrodeposition following an anodic neutralization mechanism is achievable. In addition, it has been suggested that the carboxyl and amino groups of CMCS can form hydrogen bonds with SF molecules [21]. We hypothesized that SF can cross-link with CMCS via hydrogen bonds and then electrodeposit along with CMCS on the anode to form a composite hydrogel (see Graphical Abstract).

Due to the high capacity of hydrogels for the absorption of physiological exudates from wounds and their capability to provide the wound area with a moisture balance that can enhance wound healing, hydrogels have been received increasing attention in the field of wound care [7,22,23]. Considering the intrinsic properties of both SF and CMCS as mentioned earlier, we believe a SF/CMCS composite hydrogel may exhibit a promising potential for use as a wound dressing.

In this work, we aimed to fabricate an SF-based hydrogel under low-driving voltage by exploiting the electrogelation process of CMCS and the interactions between SF and CMCS molecules in solution. The introduced strategy for SF-based hydrogel preparation does not require any additives (such as metal ions) and allows for a precisely controlled and tunable thickness, which breaks the micrometer scale limitation encountered in previous works. Subsequently, the cytotoxicity and the wound healing potential of the prepared hydrogel were investigated. It should be noted that no comprehensive work was dedicated to assess the effectiveness of the SF/CMCS composite as a wound healing agent at present.

## 2. Results

Here, an SF/CMCS hydrogel, prepared from a solution containing 5% SF and 2% CMCS under a voltage of 4 V in DC mode, was selected (the detailed explanation can be found in Section 1 of the Supplementary Materials, Figures S1–S5). The prepared hydrogel can be easily detached from the surface of the anode and independently used as a wound dressing to implement the subsequent characterization and evaluation. No hydrogel deposition was observed on the surface of the cathode or on the reference electrode.

### 2.1. Preparation of the SF/CMCS and CMCS Hydrogels

**Figure 1:**
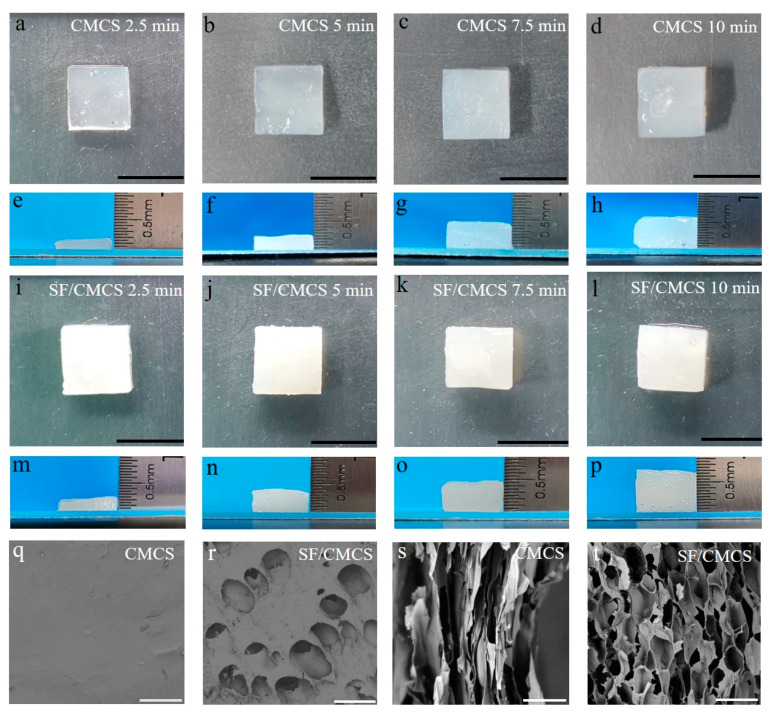
Photographs of the CMCS hydrogels prepared from 2% CMCS solution and the SF/CMCS hydrogels prepared from a 5% SF and 2% CMCS mixture with a DC voltage of 4 V applied for 2.5 min (**a**,**l**), 5.0 min (**b**,**j**), 7.5 min (**c**,**k**), and 10 min (**d**,**p**). (**e**–**h**,**m**–**p**) are the corresponding side-view images of (**a**–**d**,**i**–**l**), respectively (scale bar: 1 cm). (**q**,**r**) present the SEM images of the top surface morphology of freeze-dried CMCS hydrogel and SF/CMCS hydrogel, respectively. (**s**,**t**) exhibit the cross-sectional SEM images of CMCS and SF/CMCS hydrogels, respectively (scale bar: 200 µm).

Figure 1a–h shows cloudy white hydrogels prepared from 2% CMCS solution, and Figure 1i–p shows milky white hydrogels fabricated from a 5% SF and 2% CMCS mixture under a DC voltage of 4 V for different durations. All hydrogels exhibit a smooth and homogeneous surface, and their thickness increased with increasing the electrodeposition time. In addition, all the SF/CMCS hydrogels exhibit greater thickness than that of CMCS hydrogel. It should be noted that no silver ion leakage from the Ag/AgCl reference electrode was detected during the electrodeposition process (Supplementary Materials Section 2).

The microstructures of freeze-dried CMCS hydrogel and SF/CMCS hydrogel were investigated using a scanning electron microscope (SEM). The surface of the prepared CMCS hydrogel was obviously smooth and uniform, without a porous structure (Figure 1q), whereas SF/CMCS hydrogels demonstrated a porous surface morphology (Figure 1r). The SEM cross-sectional images for the freeze-dried CMCS hydrogels and SF/CMCS hydrogels are shown in Figure 1s,t. CMCS hydrogel was found to consist of close packed layers; this layered structure was also observed in the electrodeposition of chitosan hydrogel from previous studies [24]. SF/CMCS hydrogel demonstrates a cross-linking hierarchical porous architecture, with an average pore diameter of 98.27 ± 17.45 μm (Table S1), determined from SEM images using the analysis tool in ImageJ software (National Institutes of Health, Bethesda, MD, USA). 

According to the approach described in the Materials and Methods section, 65.2% ± 13.9% of SF protein was present in the SF/CMCS hydrogel, which indicates that SF was the main component of the SF/CMCS hydrogel. The cumulative release of SF over time was measured by immersing the SF/CMCS hydrogel in deionized water. Then, the amount of protein released was determined through a Bradford protein assay. In the first 6 h, only a negligible amount of SF (approximately 0.374%, Table 1) was released, confirming the efficient cross-linking of SF and CMCS molecules.

**Figure 2:**
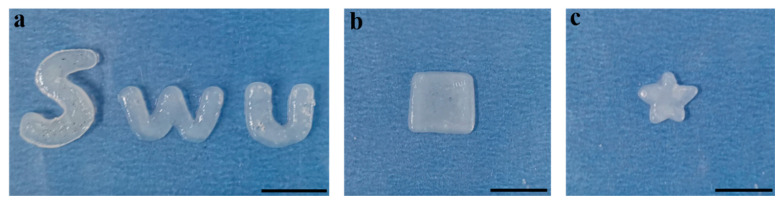
Photographs of the SF/CMCS hydrogel film with different shapes: (**a**) “SWU” logo, (**b**) square, and (**c**) pentagram (scale bar: 1 cm).

Figure 2 shows that a hydrogel film with tailored shapes was then prepared by patterning the surface of the graphite electrode through an insulating block layer. This demonstration reveals that the reported approach allows a programmable and controllable means for assembling an SF-based hydrogel with spatial selectivity.

### 2.2. Rheological Measurements

**Figure 3:**
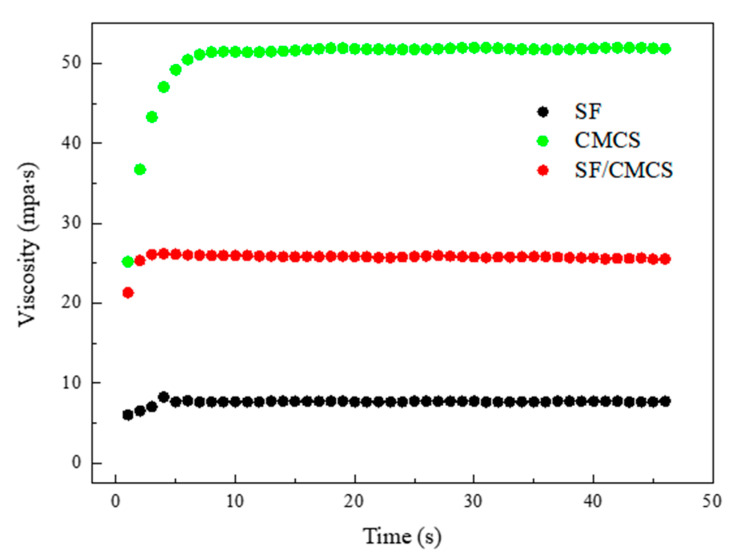
Viscosity–time curves of the SF solution, CMCS solution, and SF/CMCS mixture.

Rheological measurements were performed on the polymer solutions to assess the behaviors of SF and CMCS before and after they were mixed together. Figure 3 shows that the viscosity of the 10% SF solution was 7.70 ± 0.04 mpa·s, and the viscosity of the 4% CMCS solution was 51.77 ± 0.09 mpa·s. The viscosity increased to 25.85 ± 0.13 mpa·s upon mixing the SF and CMCS solutions together while maintaining a composition of 5% SF and 2% CMCS in the mixture.

### 2.3. Mechanical Properties and Infrared Spectroscopy (FTIR) of the SF/CMCS Hydrogel

**Figure 4:**
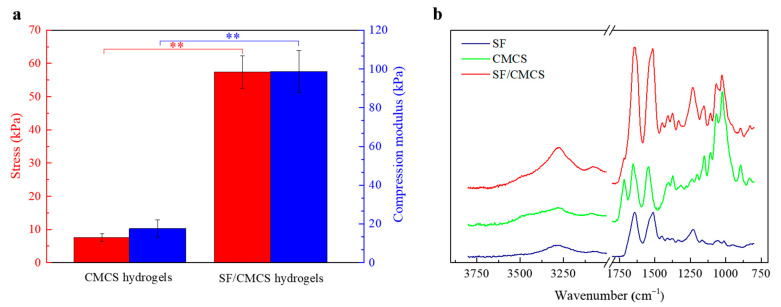
(**a**) Compression test results of the CMCS and SF/CMCS hydrogels and (**b**) FTIR spectra of the SF, CMCS, and SF/CMCS hydrogels. Statistical analysis was performed using an unpaired, two-tailed *t*-test (0.05, ** *p* < 0.01, *n* = 3).

The compressive properties of the prepared CMCS and SF/CMCS hydrogels were measured as shown in Figure 4a. According to Figure 4a, the compressive modulus and stress of the SF/CMCS hydrogel were significantly improved compared with those of the CMCS hydrogel.

An FTIR analysis of the CMCS and SF/CMCS hydrogels, as well as an SF sponge prepared through the lyophilization of SF aqueous solution, is shown in Figure 4b. The FTIR spectra of the SF/CMCS hydrogel exhibited the characteristic absorption bands of the SF sponge and the CMCS hydrogel with only an intensity difference, confirming that the SF/CMCS hydrogel is a combination of SF and CMCS.

### 2.4. Thermogravimetric Analysis (TGA)

**Figure 5:**
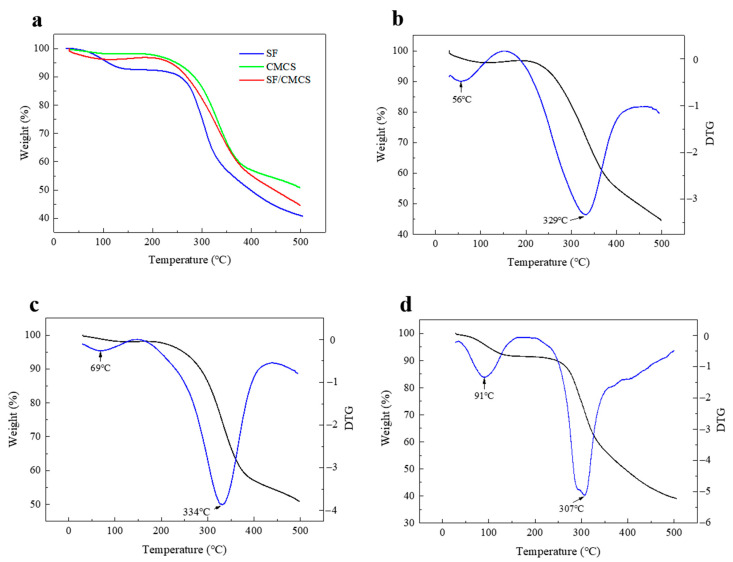
(**a**) TGA curves of the SF sponge, CMCS hydrogel, and SF/CMCS hydrogel. TGA traces (black dashed line) and the corresponding DTG thermographs (blue solid line) of (**b**) SF/CMCS hydrogel, (**c**) CMCS hydrogel, and (**d**) SF sponge.

The degradation and thermal stability behavior of lyophilized CMCS and SF/CMCS hydrogels, as well as an SF sponge prepared through lyophilization, were evaluated via TGA. The TGA curves (Figure 5a) revealed that weight loss occurred in two distinct stages. The first stage was due to the evaporation of absorbed moisture from 30 °C to 150 °C. The derivative thermogravimetric (DTG) curve of the SF/CMCS sample (Figure 5b) showed a peak water loss at 56 °C compared to 69 °C and 91 °C for the CMCS and SF samples (Figure 5c,d), respectively.

The second stage related to the degradation of the samples from 150 °C to 500 °C. The DTG peaks of the CMCS and SF samples showed a maximum value of 334 °C and 307 °C, respectively, compared to 329 °C for the SF/CMCS sample. These results are in accordance with previous findings that CMCS has better thermal stability than the regenerated silk fibroin [25,26].

### 2.5. Swelling Study and Water Vapor Transmission Rate (WVTR)

**Figure 6:**
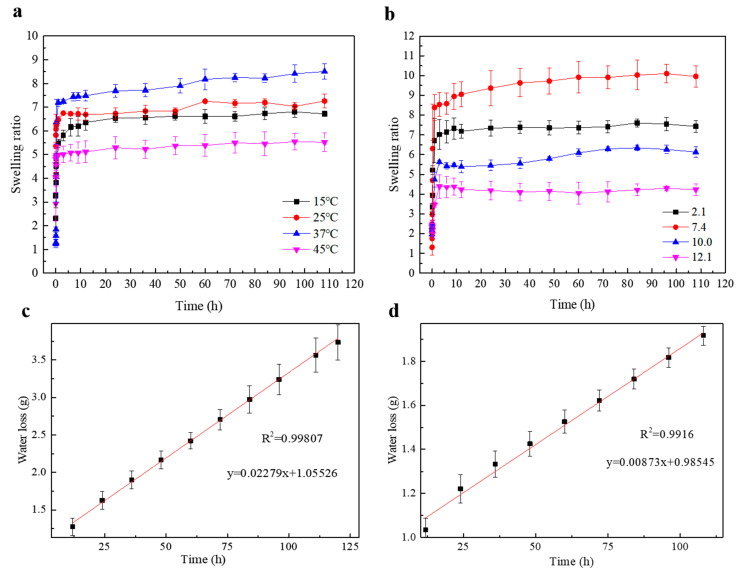
Swelling kinetics of SF/CMCS hydrogels at different temperatures (**a**) and pH values (**b**). Water vapor transmitted across the CMCS hydrogel (**c**) and SF/CMCS hydrogel (**d**).

The swelling capacity of the SF/CMCS hydrogel in response to different temperatures and pH values was assessed. Figure 6a shows the swelling kinetics of the SF/CMCS hydrogel in deionized water at 15 °C, 25 °C, 37 °C, and 45 °C. Although the testing temperatures are different, SF/CMCS hydrogels reached swelling equilibrium within the first 12 h and demonstrated remarkable swelling behavior. As the temperature increased from 15 °C to 45 °C, the equilibrium swelling ratio of the SF/CMCS hydrogel first increased then decreased. The equilibrium swelling ratio of the SF/CMCS hydrogel reached its maximum value at 37 °C and reached its minimum value at 45 °C.

To investigate the influence of pH on the swelling behavior of the SF/CMCS hydrogel, solutions under four pH levels from 2.1 to 12.1 were prepared, and the ionic strength was maintained at 0.004 M with KCl to eliminate the effect of ionic strength. Figure 6b shows the swelling kinetics of SF/CMCS hydrogels in solutions with different pH values at room temperature (25 °C). The results show that SF/CMCS hydrogels reached their swelling equilibrium within the first 12 h. Furthermore, it was found that the degree of swelling reached a maximum at pH 7.4 and decreased to a minimum at pH 12.1.

As shown in Figure 6c, the water vapor permeability of the SF/CMCS hydrogel was 2095.2 ± 68.2 g·m^−2^·day^−1^, and the CMCS hydrogel demonstrated a WVTR of 5620.0 ± 35.5 g·m^−2^ day^−1^ (Figure 6d).

### 2.6. In Vitro Stability Evaluation of the Hydrogels

**Figure 7:**
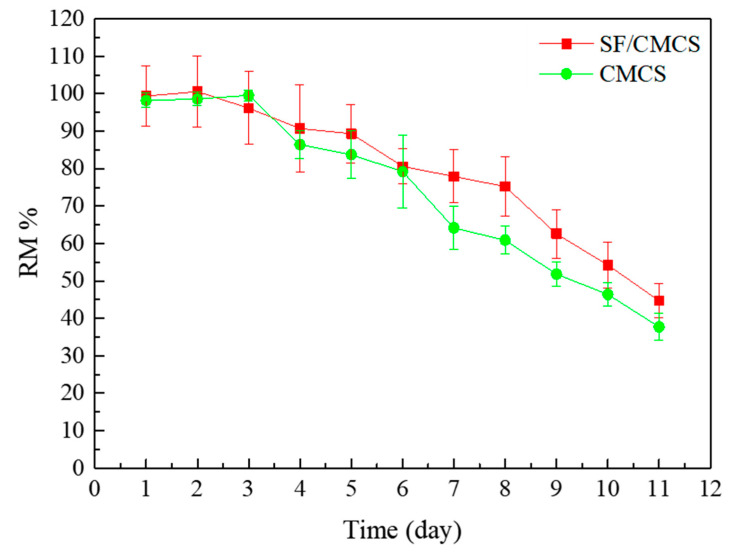
The degradation profiles of SF/CMCS and CMCS hydrogels.

It is important to understand the physical stability of the hydrogel for wound dressing. The fast degradation would result in reduced hydration or diminished mechanical stiffness of the hydrogel. In contrast, slow degradation might result in sticking to the wound, which would need to be surgically removed [27,28,29]. The degradation kinetics of both SF/CMCS and CMCS hydrogels were studied and the results are shown in Figure 7. It is obvious that the mass of both hydrogels remain constant, and the hydrogel degraded slowly after 3 days. In addition, the degradation rate of the SF/CMCS hydrogel was smaller than that of the CMCS hydrogel with 44.72% ± 4.48% and 37.74% ± 3.69% of the remaining mass (RM%), respectively.

### 2.7. Antimicrobial Activity

**Figure 8:**
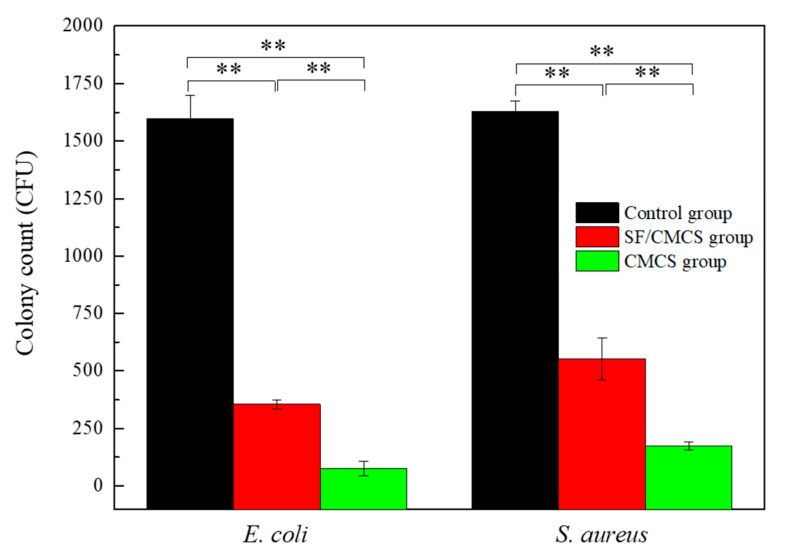
Comparison of the colony numbers of *S. aureus* and *E. coli* on different agar plates. Statistical analysis was performed using an unpaired, two-tailed *t*-test (** *p* < 0.01, *n* = 3).

The results (Figure 8) demonstrated the good antibacterial activities of SF/CMCS and CMCS hydrogels against *Escherichia coli* (*E. coli*) and *Staphylococcus aureus* (*S. aureus*), respectively, determined by the colony counting method. The representative images of agar plates for the control group, the CMCS group, and the SF/CMCS group against *E. coli* and *S. aureus* are shown in Figure S6. The antibacterial property of the hydrogel was further confirmed in Section 5 of the Supplementary Materials, Figure S7.

### 2.8. Cytotoxicity Test

**Figure 9:**
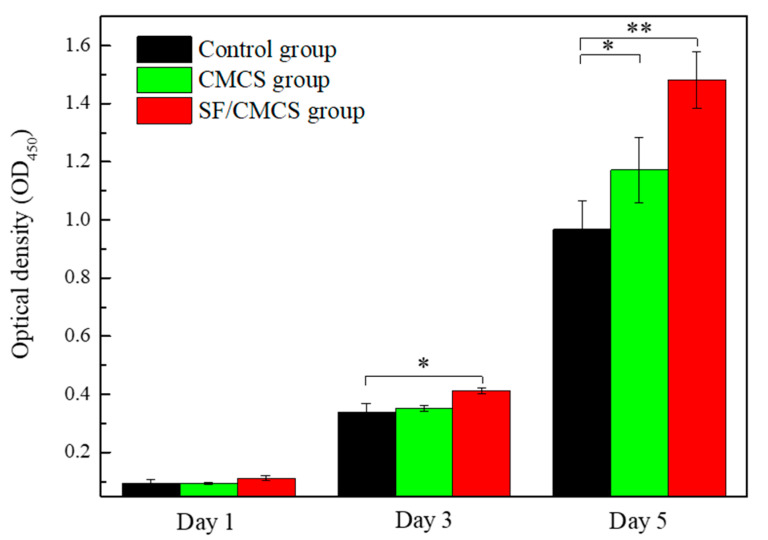
Viability of HEK-293 cells after 1 day, 3 days, and 5 days of contact with leaching liquor obtained from CMCS hydrogel and SF/CMCS hydrogel. Statistical analysis was performed using an unpaired, two-tailed *t*-test (* *p* < 0.05, ** *p* < 0.01, *n* = 3).

In this study, the cytotoxicity of the samples was assessed using the Cell Counting Kit-8 (CCK-8) assay. HEK-293 cells were chosen as the model cell because of their wide usage in toxicity evaluations for the safe use of materials [30,31,32]. The morphology of HEK-293 cells in the CCK-8 assay is shown in Figure S8. As depicted in Figure 9, cell proliferation after 3 days of culturing was assessed. The proliferation rate of the SF/CMCS hydrogel leaching liquor-treated group (121.55% ± 3.41%) was higher than that of the CMCS hydrogel leaching liquor-treated group (103.54% ± 2.99%). A similar conclusion can be drawn from the CCK-8 assay after 5 days of cell seeding: the SF/CMCS hydrogel (153.24% ± 9.98% cell proliferation rate) was more biocompatible than the CMCS hydrogel (121.17% ± 11.61% cell proliferation rate).

### 2.9. In Vivo Wound Healing Assay

**Figure 10:**
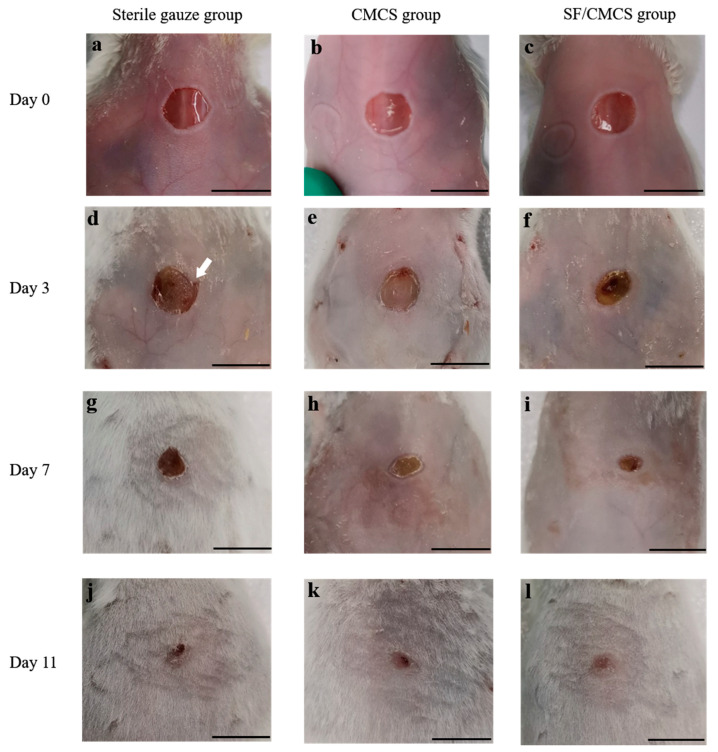
Wound healing of the sterile gauze group, CMCS group, and SF/CMCS group. (**a**,**d**,**g**,**j**) are the gross observations of wounds treated by sterile gauze at the 0th, 3rd, 7th, and 11th days, respectively. (**b**,**e**,**h**,**k**) are the gross observations of wounds treated by CMCS hydrogels at the 0th, 3rd, 7th, and 11th days, respectively. (**c**,**f**,**i**,**l**) are the gross observations of wounds treated by SF/CMCS hydrogels at the 0th, 3rd, 7th, and 11th days, respectively (scale bar: 1 cm).

To evaluate the wound healing effect of the prepared SF/CMCS hydrogel for the acceleration of wound repair, a full-thickness skin defect mouse model, created on the mouse dorsum with a biopsy punch, was investigated. Figure 10 displays the gross observations of wounds treated by sterile gauze (sterile gauze group), CMCS hydrogels, and SF/CMCS hydrogels at the 0th, 3rd, 7th, and 11th days. On day 3, the wound region displayed decay and a dark red color. Broken skin was found on the edge of the wound (indicated by the white arrow in Figure 10d), which might have been caused by the shear force from the gauze and the mouse’s skin moving. Treatment with the CMCS hydrogel slightly reduced the size of the wound region, whereas the SF/CMCS hydrogel significantly reduced the wound region, with the formation of granulation tissue. The percentage reduction in wound size of the sterile gauze, the CMCS hydrogel, and the CMCS/SF hydrogel treatment groups on day 3 were calculated using ImageJ software (Table S2) to be 6.29% ± 0.98%, 14.20% ± 6.05%, and 47.09% ± 4.09%, respectively. On the 7th day after surgery, the wound margin of all groups receded toward the wound center, in which the CMCS/SF group exhibited a better rate of wound healing, with a percentage reduction in wound size of 83.03% ± 0.66%, compared with the sterile gauze group and the CMCS group. After 11 days of treatment, the SF/CMCS group displayed almost complete wound contraction, as shown in Figure 10l.

**Figure 11:**
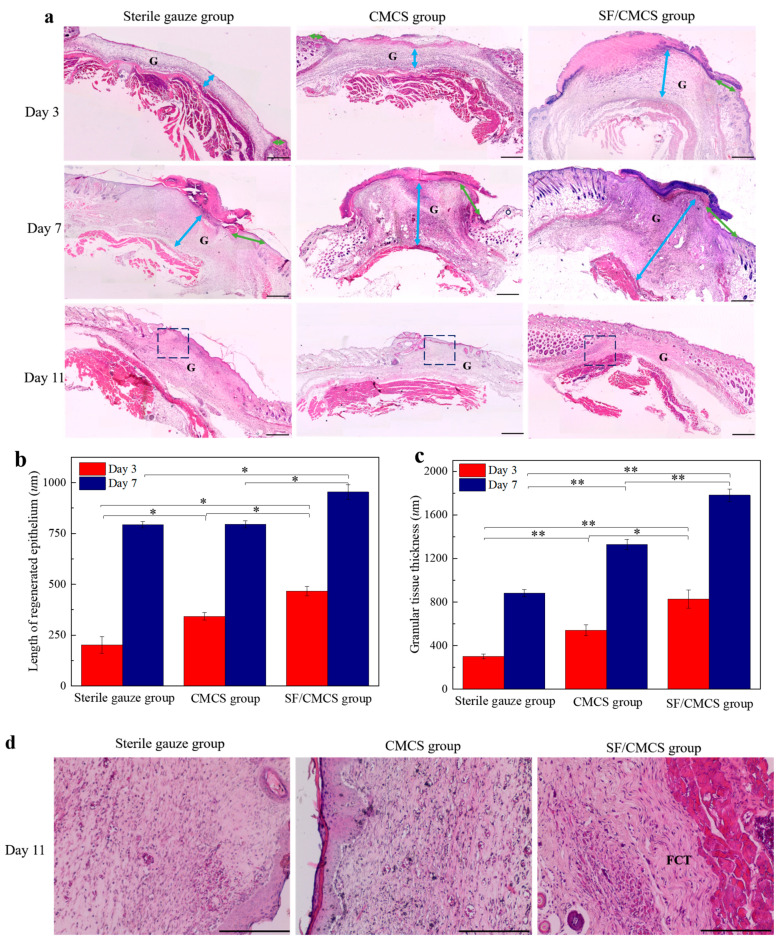
(**a**) Analysis of H&E staining in the sterile gauze group, CMCS group, and SF/CMCS group at 3, 7, and 11 days, respectively. The blue arrow represents the thickness of granulation tissue, and the green arrow represents the length of regenerated epithelium (G: granulation tissue, scale bar: 500 µm). The regenerated epithelium length (**b**) and average granulation tissue thickness (**c**) of the three groups were compared on the 3rd and 7th days. Statistical analysis was performed using an unpaired, two-tailed *t*-test (* *p* < 0.05, ** *p* < 0.01, *n* = 3). (**d**) The granulation tissue of the three groups was observed at 11 days, corresponding to the boxed regions in (**a**) (FCT: fibrous connective tissue, scale bar: 200 µm).

On the basis of hematoxylin and eosin (H&E) stained slides, the length of the newly formed epithelium was measured using ImageJ software (ImageJ 1.6.0) and is indicated by green arrows in Figure 11a. As shown in Figure 11a,b, on the third day after surgery, the length of the newly formed epithelium in the SF/CMCS group (466.61 ± 23.99 µm) was longer than those in the CMCS group (342.56 ± 18.86 µm) and the sterile gauze group (201.27 ± 40.58 µm). The groups showed a similar tendency on the 7th day, with a length of 953.73 ± 36.76 µm for the SF/CMCS group, 795.22 ± 17.80 µm for the CMCS group, and 793.16 ± 16.27 µm for the sterile gauze group.

The thickness of the granulation tissue of all samples was measured using IPP software (Image-Pro Plus 6.0, Media Cybernetics, Rockville, MD, USA) and is marked by the blue arrows in Figure 11a. On day 3 and day 7, the SF/CMCS group showed significantly thicker granulation tissue than both the CMCS group and the control group, of which the control group had the lowest granulation accumulation.

On the 11th postoperative day, the wounds of all groups were epithelialized. The H&E section of the SF/CMCS group, as depicted in Figure 11d, shows fibrous connective tissue (FCT) with regularity, but no fibrous connective tissue was observed in the H&E section of the sterile gauze and CMCS groups.

## 3. Discussion

The thickness of the CMCS and SF/CMCS hydrogels gradually increased with electrodeposition time (Figure 1a–p), which is consistent with the observations of other studies on hydrogel preparation via electrodeposition [10,18,19]. It can be observed that the thickness of SF/CMCS hydrogels is greater than that of CMCS hydrogels prepared under identical conditions. These results indicate that cross-linking interactions occurred in the SF/CMCS solution.

The SEM observations (Figure 1q–t) found that the SF/CMCS hydrogel had a cross-linked structure with a hierarchical porous architecture, which is obviously different from the layered structure of the CMCS hydrogel. This result might be due to the prevention of sheet-structure formation by means of the interaction between SF and CMCS. It has been reported that hydrogels with a porous structure help to transport oxygen to the wound, absorb wound exudate, and balance the moisture level at the wound site [33,34]. Hence, compared to CMCS hydrogels, SF/CMCS hydrogels exhibit superior characteristic features in terms of the physical structures of wound dressings. In addition, the higher crosslinking network of the SF/CMCS hydrogel might be the reason for its higher stability compare to that of the CMCS hydrogel.

The viscosity of a mixture of two fluids can be determined according to the Arrhenius equation [35] as follows:

(1) $$ln\;\eta_{m}=\phi_{1}ln\;\eta_{1}+\phi_{2}ln\;\eta_{2}$$

where $\eta_{m}$ is the viscosity of fluid mixture, $\phi_{i}$ is the volume fraction of component *i*, and $\eta_{i}$ is the viscosity of component *i*.

The theoretical viscosity of the prepared SF/CMCS mixture can be calculated as 19.96 mpa·s, which is smaller than the experimental data, indicating that some interaction has occurred between SF and CMCS. Because the carboxyl groups (pKa = 4.5) [36] of CMCS and SF (pI = 4.5) [37] are predominantly negatively charged at the pH of the mixture (pH = 8.76), the formation of an electrostatic interaction between SF and CMCS is less favorable. We believe this difference in the calculated theoretical and experimental viscosities of the SF/CMCS mixture was mainly due to the formation of hydrogen bonds between SF and CMCS, as the rheological behavior of an aqueous polymer mainly depends on the relative strength of the hydrogen bonding within the solution, and more strong hydrogen bonds would greatly increase the viscosity [38,39].

The compression test results (Figure 4) reveal that the SF/CMCS hydrogel possessed enhanced compressive mechanical properties compared with the CMCS hydrogel, which can be attributed to the interaction between SF and CMCS molecules. As mechanical properties can reflect structural stability [10], the results demonstrate that the electrodeposition of the SF/CMCS mixture has a better gel-forming effect.

The TGA curves shown in Figure 5 indicate that water was loosely held by the SF/CMCS sample and easily removed at a lower temperature, compared to the water physically entrapped by SF and CMCS. This is because most hydrophilic groups from SF and CMCS are already involved in hydrogen bonding, therefore exhibiting a lower moisture absorption level [40]. As shown in Figure 5a, a slow, continuous mass loss was observed in the TGA curve of the SF/CMCS sample without matching the TGA curves of SF and CMCS. This result suggests a considerable interaction between SF and CMCS [41]. The SF/CMCS sample exhibited lower thermal stability than the CMCS sample, possibly because the introduction of RSF decreased the crystallinity of CMCS. This trend in the thermal stability of the samples is supported by the mass of the remaining samples (Figure 5a).

Swelling is an important feature of hydrogel dressings. Large water absorption capacities allow hydrogel dressings to absorb wound fluids and exudates [42]. The equilibrium swelling ratio of the SF/CMCS hydrogel first increased and then decreased with temperature (Figure 6a). This trend can be explained in terms of H-bonding within the hydrogel, which was weakened by the larger thermal motion at higher temperatures. This H-bond weakening loosens the hydrogen bond cross-links, enlarging the equilibrium swelling ratio. As the temperature was further increased to 45 °C, the equilibrium swelling ratio of the SF/CMCS hydrogel decreased dramatically, caused by hydrogel dissociation due to the breaking of hydrogen bonds.

The surface pH of a wound varies [43]. The influence of pH on the swelling behavior of the SF/CMCS hydrogel was evaluated. The swelling equilibrium state of SF/CMCS hydrogels in solution is pH-dependent, which can be largely attributed to the ionization/deionization processes of pH-sensitive functional groups. In an acidic environment (pH 2.1), carboxylic groups (-COO^−^) introduced by carboxymethyl chitosan were protonated as -COOH, resulting in a stronger hydrogen-bonding interaction. Consequently, the hydrogel network shrank and the swelling equilibrium decreased. When the pH values increased to seven, the carboxyl group was ionized, and the electrostatic repulsion between molecular chains became dominant, leading to an expansion of the hydrogel network [44]. However, when the pH values continued to increase to 10.0 and 12.1, Na^+^ and K^+^ in the solution combined with the -COO^−^ groups of the SF/CMCS hydrogel (Na^+^ and K^+^ were introduced when preparing the buffer solutions with pH values of 10.0 and 12.1; see Section 3 of the Supplementary Materials). This charge shielding neutralized the electrostatic interactions and reduced the electrostatic repulsion. The gel network therefore shrank into a compact structure with a decreased swelling equilibrium [45,46]. The above experiments prove that the prepared SF/CMCS hydrogels are pH- and temperature-sensitive, with the capability to absorb exudate in the wound environment.

An ideal wound dressing can control water vapor loss from a wound at an optimal range (2000–2500 g·m^−2^·day^−1^) to avoid either excessive dehydration or exudate accumulation at the wound region [47]. Based on the obtained WVTR data, it was found that the SF/CMCS hydrogel, with a WVTR of 2095.2 ± 68.2 g·m^−2^·day^−1^, could maintain a suitable moisture environment for wound healing; whereas the CMCS hydrogel, with a WVTR of 5620.0 ± 35.5 g·m^−2^·day^−1^, may result in dehydration or slow healing of the wound area. The hydrogen bonding between SF and CMCS presumably weakens their hydrophilicity, consequently reducing the water vapor transmission rate of the SF/CMCS hydrogel [48]. Compared to the CMCS hydrogel, the SF/CMCS hydrogel is a more suitable wound dressing that can create environments that are supportive of earlier healing outcomes.

Wound infections caused by bacteria could cause either a delay in wound healing or deterioration of the wound [49,50,51]. Therefore, an ideal wound dressing should have broad-spectrum antibacterial activity [34]. SF/CMCS hydrogels exhibit better antibacterial activity than the control group. Their rates of bacteriostasis against *E. coli* and *S. aureus* were 77.73% ± 1.24% and 66.08% ± 5.54%, respectively. CMCS hydrogels exhibited much better bacteriostasis rates against *E. coli* (95.21% ± 1.94%) and *S. aureus* (89.37% ± 1.03%) than the SF/CMCS group. Although CMCS possesses excellent antibacterial properties [52], silk fibroin has little or no inherent antibacterial properties [53,54], Compared to the CMCS hydrogel, the presence of silk fibroin lowers the content of CMCS within the SF/CMCS hydrogel under the same mass weight, resulting in a weaker antibacterial performance.

In addition to antibacterial properties, biocompatibility is another inherent property that should be evaluated to determine whether SF/CMCS hydrogels are qualified to serve as wound dressings. Results of the CCK-8 assay (Figure 9) suggested that the prepared SF/CMCS hydrogels were biocompatible and showed greater cell proliferation than CMCS hydrogels, although CMCS hydrogels can also promote cell proliferation. This property is due to the presence of the Arg-Gly-Asp sequence in the silk fibroin, which is effective in promoting cell proliferation [55,56].

Gross wound observations (Figure 10) showed that the prepared SF/CMCS hydrogel can induce wound contraction and accelerate wound closure.

The four phases of wound healing include hemostasis, inflammation, proliferation, and maturation [33], and can be determined by the length of newly formed epithelium and the thickness of the granulation tissue [57,58]. The length of newly formed epithelium can be defined as the distance between the tip of the migrating keratinocytes and the first hair follicle observed on the side of the wound margin [59,60]. Wound re-epithelialization can prevent infection and excessive moisture loss [61]. These results, relating to the regenerative epithelium length, indicate that the SF/CMCS hydrogel was able to promote wound healing via wound re-epithelialization, as compared with the CMCS group and the sterile gauze group.

Histologically, granulation tissue appears bright red and granular. It is a collection of small, microscopic blood vessels and is characterized by the proliferation of fibroblasts, accompanied by inflammatory cell infiltration [62]. Granulation tissue plays an important role in the process of wound healing and fill wounds, conferring protection from infection and further damage [63]. On days 3 and 7, the granulation tissue thickness in the SF/CMCS group was substantially greater than those of the other groups (Figure 11c), and there was obviously greater granulation tissue formation in the CMCS group than in the sterile gauze group. Therefore, SF/CMCS and CMCS hydrogels appear to accelerate wound healing during the granulation phase, but SF/CMCS hydrogels exhibited superior wound healing compared to that of CMCS hydrogels.

As the granulation tissue began to mature, the wound area was filled with aligned fibrous connective tissue, which contributes to the formation of new tissue and subsequent tissue remodeling [64,65]. This transformation could be seen on the 11th day of wound healing in mice treated with the SF/CMCS hydrogel, whereas it was not observed in the CMCS group or in the sterile gauze group, indicating that the SF/CMCS hydrogel could facilitate the formation of fibrous connective tissue for wound repair.

The above results indicate that SF/CMCS hydrogels could promote wound healing by favoring granulation tissue formation, wound re-epithelialization, and the conversion of granulation tissue into fibrous connective tissue. The promising wound healing properties of the SF/CMCS hydrogel are consistent with other experimental results showing that SF/CMCS hydrogels can provide a good barrier to prevent dehydration of the wound site and accelerate cell proliferation. The wound healing-promoting characteristics of the SF/CMCS hydrogel can also be attributed to the presence of the inherent tripeptide sequence of arginine-glycine-aspartic acid (RGD) in the protein fibroin sequences, which can promote cell adhesion and migration [66]. To the best of our knowledge, this is the first time that the therapeutic effect of an SF/CMCS composite has been evaluated in wound healing.

## 4. Materials and Methods

### 4.1. Materials and Reagents

Cocoons of silkworm *Bombyx mori* (a Chinese strain demoted as 872) were provided by College of Biotechnology, Southwest University, China. Carboxymethyl chitosan (CMCS, pale yellow powder, viscosity ≤50 mpa·s, and degree of substitution ≥95.0%) was purchased from Shanghai Ryon Biological Technology CO., Ltd. (Shanghai, China). Sodium carbonate, sodium chloride, aminoacetic acid, potassium chloride, boric acid, xylene, potassium dihydrogen phosphate, and disodium hydrogen phosphate dodecahydrate were purchased from KeLong Chemical Reagent Co., Ltd. (Chengdu, China). Calcium chloride was purchased from Yuanye Bio-Technology Co., Ltd. (Shanghai, China). Anhydrous ethanol and hydrochlorc acid (36.0–38.0%) were purchased from Chuandong Chemical Co., Ltd. (Chongqing, China). Potassium bromide was purchased from Sangon Biotech Co., Ltd. (Shanghai, China). Bradford protein assay kit was purchased from Beyotime Biotechnology Co., Ltd. (Shanghai, China). Sodium hydroxide was purchased from Jinshan Chemical Test Co., Ltd. (Chengdu, China). Tryptone was purchased from Beijing Aoboxing Bio-tech Co., Ltd. (Beijing, China). Yeast extract was purchased from Oxoid Co., Ltd. (Basingstoke, Britain). Trichloroacetaldehyde hydrate and formaldehyde solution were purchased from Shanghai Aladdin Bio-Chem Technology Co., Ltd. (Shanghai, China). A Cell Counting Kit-8 (CCK-8) was purchased from Mei5 Biotechnology Co., Ltd. (Beijing, China). Hematoxylin and eosin stain solution was purchased from Nanchang Yulu Experimental Equipment Co., Ltd. (Nanchang, China). All chemicals were of analytical grade and were used without further purification. Deionized water was obtained from a Milli-Q Direct-8 purification system (resistivity >18 MΩ cm, Millipore Inc., Boston, MA, USA) onsite and was used in all experiments.

### 4.2. Fabrication of SF/CMCS Hydrogel

#### 4.2.1. Preparation of RSF Solution

Cocoons from *Bombyx mori* were cut into pieces, boiled for 30 min in an aqueous solution of 0.5% Na_2_CO_3_, and rinsed with water to extract sericin. This operation was repeated twice. Then, the degummed silk fiber was dissolved in a ternary solvent of CaCl_2_:CH_3_CH_2_OH:H_2_O, in a molar ratio of 1:2:8, at 70 °C until the silk fibroin was completely dissolved. Once the silk fibroin salt solution was cooled to room temperature, it was dialyzed (MWCO 8000, Solarbio, Beijing, China) against deionized water for 3 days, changing the water every 2 h. After dialysis, the silk fibroin solution was filtered through gauze and centrifuged at 4 °C and 8000 rpm to remove silk aggregates and debris. The resulting RSF solution obtained was concentrated at 60 °C in a water bath to prepare silk fibroin solutions with different mass fractions. 

#### 4.2.2. Preparation of CMCS Solution

The CMCS solution was prepared by dissolving CMCS in deionized water, and the pH was adjusted with 5 M sodium hydroxide to 12 for complete dissolution [67]. Our experiments suggest that 4% is the highest mass/volume ratio for maintaining CMCS aqueous solution in a stable state without precipitation.

#### 4.2.3. Preparation of SF/CMCS Hydrogel

SF solution of a certain mass fraction was blended with 4% CMCS solution for 4 h at 250 rpm at room temperature (25 °C) to prepare the desired SF/CMCS mixture. It has been reported that low temperature benefits hydrogen bond formation [68]. Therefore, to facilitate the formation of intermolecular hydrogen bonds between SF and CMCS, the SF/CMCS mixture was kept in a refrigerator at 4 °C overnight.

An electrochemical workstation (CHI760E, Shanghai Chenhua Instruments Limited, China) and a three-electrode assembly were used for the SF/CMCS hydrogel fabrication using a graphite anode, an Ag/AgCl reference electrode, and a platinum cathode. The electrodes were immersed in SF/CMCS aqueous solution, which contained 0.25% (*w*/*v*) NaCl as electrolyte, and a voltage in direct current (DC) mode was applied over a period of time by means of chronoamperometry. The generated hydrogel was gently peeled from the graphite electrode and then carefully rinsed with deionized water to remove residue.

### 4.3. Evaluation of SF Content in SF/CMCS Hydrogel

The percentage of SF (*n%*) within the prepared SF/CMCS hydrogel was measured based on the Bradford protein assay [69] using the following equation:

(2) $$n\%=\frac{C_{1}V_{1}-C_{2}V_{2}}{m}\times 100\%$$

where $C_{1}$ is the concentration of SF in the SF/CMCS mixture before gelation, and $C_{2}$ is the concentration of SF in the remaining solution after gelation. $C_{1}$ and $C_{2}$ can be determined from the standard calibration curves presented in Figure S9a and S9b, respectively. m is the mass of the prepared SF/CMCS hydrogel after lyophilization. $V_{1}$ is the volume of the SF/CMCS mixture before gelation and is 20 mL; and $V_{2}$ is the volume of the remaining solution after gelation. Therefore, $C_{1}V_{1}$ is the content of SF in the 20 mL of SF/CMCS mixture, $C_{2}V_{2}$ is the content of SF in the remaining solution after the SF/CMCS hydrogel is prepared, and $C_{1}V_{1}-C_{2}V_{2}$ represents the mass of SF in the SF/CMCS hydrogel.

### 4.4. Quantifying the SF Release

The prepared SF/CMCS hydrogel was placed in a weighing bottle containing 20 mL (*V*) of deionized water at 25 °C with a relative humidity of 75%. At different time intervals (t = 10 min, 30 min, 1 h, 3 h, and 6 h), the content of SF in the deionized water was measured via the Bradford protein assay [69], recorded as *C*. Then, the SF/CMCS hydrogels were lyophilized and weighed, which was recorded as *m*. The percentage of SF released from the SF/CMCS hydrogel (*Q%*) can be calculated using the following formula, in which *n%* is the percentage of SF in the SF/CMCS hydrogel, determined from Formula (3):

(3) $$\;Q\%=\frac{C\times V}{\left(m+C\times V\right)\times n\%}\times 100\%\;$$

### 4.5. Characterization of the Prepared SF/CMCS Hydrogel

#### 4.5.1. Micromorphological Characterization

The samples were first freeze-dried using a lyophilizer (LGJ-10, Shanghai YuMing Instrument Co., Ltd., Shanghai, China). Subsequently, the lyophilized samples were sputter-coated (GSL-1100X-SPC-16m, MTI Corporation, Richmond, VA, USA) with gold to give them a conductive coating. The surface and cross-section morphologies of SF/CMCS and CMCS hydrogels were observed using a scanning electron microscope (Phenom Pro, Phenom-World, Eindhoven, The Netherlands).

#### 4.5.2. Rheological Characterization

Rheological measurements of the solutions were performed using a viscometer (DV3TLVCJ0, Brookfield, Middleboro, MA, USA) with a 48-mm-diameter parallel plate at room temperature (25 °C). All samples were left at rest for 5 min to allow for temperature equilibration.

#### 4.5.3. Compressive Strength Testing

The prepared hydrogels were equilibrated in deionized water for 24 h to fully swell. The compressive strength was measured using a universal testing machine (Shanghai Xieqiang Instrument Technology Co., Ltd., Shanghai, China) at a test speed of 1 mm/min.

#### 4.5.4. Fourier-Transform Infrared Spectroscopy (FTIR) Analysis

The freeze-dried hydrogel samples were ground into powder for attenuated total reflection Fourier transform infrared spectroscopy (ATR-FTIR) analysis (Thermo Scientific Nicolet iN10, Waltham, MA, USA) to determine their chemical/structural properties. The absorption spectra of samples in the mid-infrared region (4000–400 cm^−1^) were acquired at a resolution of 4 cm^−1^ and 32 scans of each sample.

#### 4.5.5. Thermogravimetric Analysis (TGA)

The thermal behaviors of the samples were studied using a simultaneous thermal analyzer (STA 449 F3 Jupiter, NETZSCH, Selb, Germany). First, the samples were lyophilized and ground into powders. Then, 5 mg of each sample was loaded in an Al_2_O_3_ crucible. The operation was performed from 30 °C to 500 °C at a constant heating rate of 10 °C/min in N_2_ atmosphere.

#### 4.5.6. Swelling Studies

Hydrogel swelling is an important feature of wound dressings that allows hydrogel dressings to absorb wound fluids and exudates [42]. Hence, the swelling kinetics of SF/CMCS hydrogels at various temperatures (15 °C, 25 °C, 37 °C, and 45 °C) and pH conditions (pH = 2.1, 7.4, 10.0, and 12.1) were evaluated to investigate the response of the hydrogels to different wound environments. SF/CMCS hydrogels were lyophilized and pre-weighed before immersion in a swelling medium. At specific intervals, the samples were removed from the swelling medium and weighed after the excess surface liquids were removed by blotting with Kimwipes. The swelling ratio (*SR*) can be determined using the following equation [70]:

(4) $$SR=\frac{m_{s}-m_{1}}{m_{1}}\times 100\%\;$$

where $m_{s}$ is the weight of the swollen hydrogel at time t, and $m_{1}$ is the weight of hydrogel after lyophilization. The experiments were performed in triplicate, and the average of the results was reported with the standard deviation.

#### 4.5.7. Measurement of Water Vapor Transmission Rate (WVTR)

The moisture vapor permeability of the hydrogel was described via the WVTR, assessed according to the method of the American Society for Testing and Materials (ASTM) [71]. The test was conducted at 37 °C and with a relative humidity of 35% in an artificial environment chamber. The prepared hydrogel was used to seal the weighing bottle, containing 10 mL of deionized water, with the effective transfer area (recorded as *A*). The bottles were weighed every 12 h to construct a plot of the rate of water loss per unit time. Its slope was then used to determine the WVTR using the following equation [63]:

(5) $$WVTR\left(g/m^{2}/day\right)=\frac{slope\times 24}{A}$$

#### 4.5.8. Degradation Study In Vitro

To investigate the stability of the hydrogel, each hydrogel was soaked in 20 mL of sterilized phosphate buffer solution (PBS, pH 7.4) until it reached a swelling balance. Every 24 h, the hydrogels were removed from the immersion solution and weighed after blotting with Kimwipes. The hydrogel degradation is expressed as the percentage of mass remaining (*RM* %), which can be calculated according to the equation:

(6) $$RM\;\%=\frac{m_{t}}{m_{0}}\times 100\%$$

where $m_{0}$ is the weight of the hydrogel that achieved balance and $m_{t}$ is the weight of hydrogel at a predetermined time point.

### 4.6. Antimicrobial Activity

The antibacterial activities of the SF/CMCS and CMCS hydrogels toward Gram-positive *Staphylococcus aureus* (*S. aureus*, ATCC 25923) and Gram-negative *Escherichia coli* (*E. coli*, ATCC 25922) were evaluated via the colony counting method [72]. To this end, 1.0 g of sterilized SF/CMCS hydrogel or CMCS hydrogel was immersed in 1.0 mL of Luria–Bertani (LB) broth with bacterial counts of 10^4^ CFU/mL, as the experimental groups. A 1.0 mL aliquot of this LB broth without an immersed sample acted as the control. After 2 h of incubation at 37 °C, the suspension was diluted 3-fold with sterilized LB solution and then subjected to the plate counting method.

### 4.7. Cytotoxicity Assay

The degree of cytotoxicity of the SF/CMCS hydrogel was assessed by measuring the cell viability of HEK-293 cells using a Cell Counting Kit-8 (CCK-8) [73]. In short, a lyophilized sample with a weight of 0.2 g was submerged in 10 mL of Dulbecco’s modified Eagle’s medium (DMEM) for 72 h at 37 °C to obtain its leaching liquor. HEK-293 cells were seeded in a 96-well plate with a density of 2 × 10^3^ cells/well in a 100-µL suspension of complete growth medium (90% DMEM with 10% FBS) and were cultured for 24 h at 37 °C in a humidified 5% CO_2_ atmosphere. Then, 10 µL of complete growth medium (control) or the leaching liquor of the samples was added to each well and incubated with cells for 24 h, 72 h, and 120 h, respectively. Subsequently, 10 µL of CCK-8 was added to each well and incubated with cells for 2 h at 37 °C. Absorbance was measured at 450 nm using a microplate reader (Synergy H1, Bio-Tek, Winooski, VT, USA). The cell proliferation rate was then calculated using the following equation:

(7) $$Cell\;proliferation\;rate\left(\%\right)=\frac{OD_{450}\left(sample\right)}{OD_{450}\left(control\right)}\times 100\%$$

### 4.8. In Vivo Wound Healing Assay

The wound healing capacity of the SF/CMCS hydrogel was evaluated using a mouse full-thickness wound defect model. Healthy, pathogen-free female Kunming mice (purchased from Tengxin Biotechnology Co., Ltd., Chongqing, China) weighing 18–22 g were anesthetized, and a round, full-thickness wound with a diameter of 6 mm was created on the dorsal side of the mouse using a skin biopsy punch. The wound was covered with SF/CMCS hydrogel (SF/CMCS group), CMCS hydrogel (CMCS group), or a sterilized gauze (Sterile gauze group). The dressings were then fixed with sutures. All procedures of animal experiments were performed in compliance with the protocols approved by the Laboratory Animal Ethics Committee of Southwest University (approval code: IACUC-20191215-17).

#### 4.8.1. Measurement of Wound Closure

The wound size of each group was recorded using a 48-megapixel camera (Sony IMX586, Tokyo, Japan) on the 3rd, 7th, and 11th days after surgery. The area of the wound was measured using ImageJ software (1.6.0, National Institutes of Health, Bethesda, MD, USA). The degree of wound closure was calculated according to the following equation [33]:

(8) $$Degree\;of\;wound\;closure\left(\%\right)=\frac{A_{0}-A_{t}}{A_{0}}\times 100\%$$

where $A_{0}$ is the initial wound area on day 0, and $A_{t}$ is the wound area on a specific day indicated after surgery.

#### 4.8.2. Histological Analysis of Wounds

Hematoxylin and eosin (H&E) staining was used for the histological analysis of wounds. Once the mice were euthanized, the wound tissue was excised and bisected. The skin wound tissue specimens were then fixed in 10% neutral-buffered formalin solution and embedded in paraffin. The tissue blocks were serially sectioned perpendicular to the skin surface at a 5-µm thickness, followed by H&E staining for histological analysis [74].

### 4.9. Statistical Analysis

All values are reported as the mean ± standard deviation for at least three replicates. Statistical analyses were performed using an unpaired, two-tailed *t*-test (* *p* < 0.05, ** *p* < 0.01).

## 5. Conclusions

In summary, relying on the electrodeposition technique, we developed an easy, green, controllable, and low-voltage approach to SF-based hydrogel fabrication by exploiting intermolecular hydrogen bonding between SF and CMCS. The prepared SF/CMCS hydrogel featured a porous architecture, pH- and temperature-sensitive equilibrium swelling behavior, antibacterial activity, and was shown to improve cell growth. According to an in vivo assessment of a full-thickness skin defect model in mice, the SF/CMCS hydrogel exhibited prominent wound healing efficiency with respect to wound re-epithelization and granulation tissue formation. These findings have significant implications for the feasibility of the developed implementation strategy for SF-based hydrogel preparation and its application as a wound dressing.

## Figures and Tables

**Table 1:** The mass percentage of cumulative SF released from the SF/CMCS hydrogel over time.

Time	10 min	30 min	1 h	3 h	6 h
Percentage (%)	0.295 ± 0.023	0.322 ± 0.010	0.325 ± 0.006	0.352 ± 0.010	0.374 ± 0.022

## Data Availability

The data presented in this study are available on request from the corresponding author.

## Supplementary Materials

The following are available online at https://www.mdpi.com/article/10.3390/ijms22147610/s1.
