# Supplementary material for: Preparation of Silk Fibroin/Carboxymethyl Chitosan Hydrogel under Low Voltage as a Wound Dressing
Source: Int J Mol Sci. 2021 Jul 16;22(14):7610. doi: 10.3390/ijms22147610 (PMC8307387; doi:10.3390/ijms22147610)
Supplement: Supplementary file 1 [file ijms-22-07610-s001.zip › ijms-1251273-supplementary.pdf]

Supplementary Materials

Preparation of Silk Fibroin/Carboxymethyl Chitosan Hydrogel under Low Voltage as a Wound Dressing

Zhenyu Chen, Xiaoning Zhang *, Jianwei Liang, Yansong Ji, Yuqing Zhou and Hao Fang

State Key Laboratory of Silkworm Genome Biology, College of Sericulture, Textile and Biomass Sciences, Southwest University, Chongqing 400715, China; c618@email.swu.edu.cn (Z.C.); liangjw@email.swu.edu.cn (J.L.); jys2020@email.swu.edu.cn (Y.J.); zhouxguai@163.com (Y.Z.); fangritian@email.swu.edu.cn (H.F.)

* Correspondence: xzhang@swu.edu.cn; Tel.: +86-15922815612

Table of Contents:

1. Optimization of gelation ……………………………………………………………………S2
2. Determination of silver ion leakage from Ag/AgCl reference electrode……………………S4
3. Preparation of buffer solutions with different pH values for swelling study ………………S5
   3.1 Preparation of amino acetic acid-hydrochloric acid buffer, pH 2.1……………………S5
   3.2 Preparation of saline (phosphate-buffered), pH 7.4 ……………………………………S5
   3.3 Boric acid-potassium chloride-sodium hydroxide buffer, pH 10.0………………………S5
   3.4 Disodium hydrogen phosphate-sodium hydroxide buffer solution, pH 12.1……………S5
4. Representative results for antibacterial test ………………………………………………S5
5. Confirmation of antibacterial property of the hydrogel……………………………………S6
6. Morphologies of HEK-293 cells in CCK-8 assay …………………………………………S7
7. Degree of wound closure ……………………………………………………………………S7
8. Standard calibration curves of SF under different conditions ……………………………S7
9. References……………………………………………………………………………………S9

1. Optimization of gelation

As shown in Figure S1 a and b, the hydrogel generated on the anode surface is too thin to be peeled off when the applied voltages were 1 V and 2 V. However, when the applied voltage increased to 6 V, the hydrogel generated exhibited a rough structure and visible pores on the surface (Figure S1c). We believe those pores are due to the oxygen bubbles produced on the graphite electrode by the following reaction [1]:

$$2H_2O(l) \rightarrow O_2(g) + 4H^+(aq) + 4e^-$$

Initially, small oxygen bubbles randomly appeared at the surface of the graphite electrode. However, nearby oxygen bubbles fused to form large bubbles.

No hydrogel was found on the surface of either the platinum electrode (cathodic electrode) or Ag/AgCl electrode (reference electrode) in all cases (Figure S1d,e).

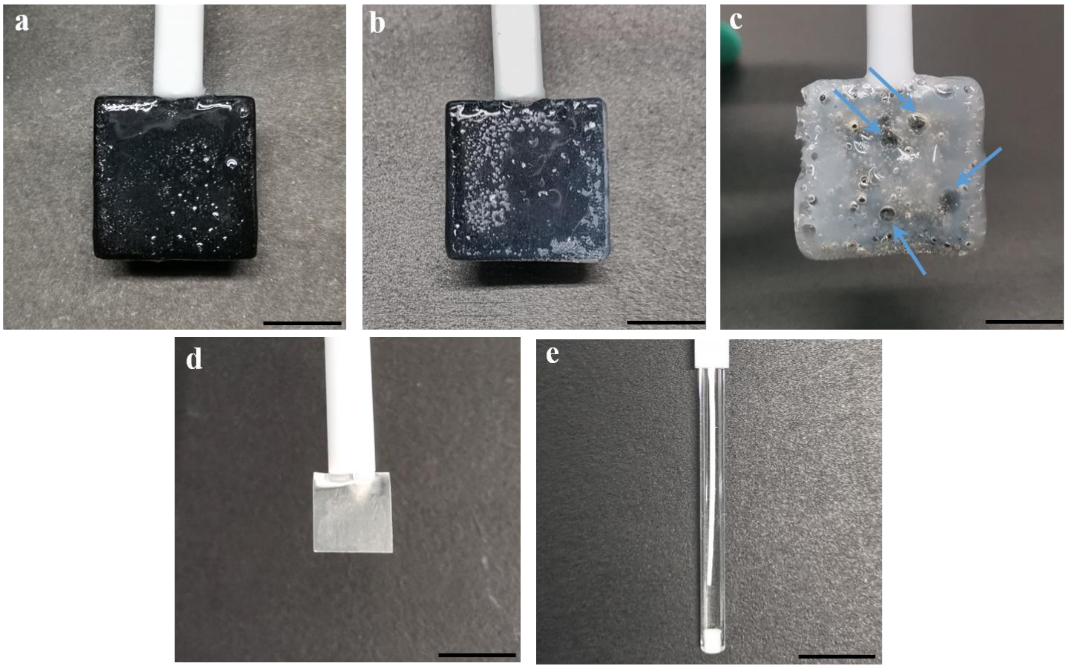

Figure S1. Photographs of SF/CMCS hydrogels electrodeposited on the graphite electrodes, from the mixture of 5% SF and 2% CMCS solution, with applied voltages of 1 V (**a**), 2 V (**b**), and 6 V (**c**) for 7.5 min. The blue arrows indicate enlarged pores. No hydrogel was observed on the platinum (Pt) electrode (**d**) and Ag/AgCl electrode (**e**) (scale bar: 1 cm).

SF/CMCS hydrogels exhibited a larger compressive stress when prepared under a direct current (DC) voltage of 4 V rather than 3 V or 5 V (Figure S2).

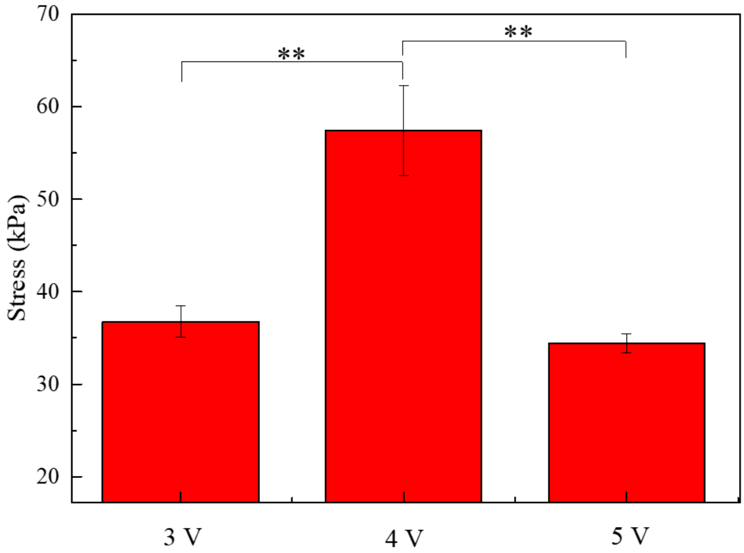

Figure S2. Compression test results of SF/CMCS hydrogels prepared from the 5% SF and 2% CMCS mixture under different voltages. Statistical analysis was performed using an unpaired, two-tailed t-test (* $p < 0.05$, ** $p < 0.01$, $n = 3$).

As the surface charge density increased with the applied voltage [2], the density of CMCS molecules deposited on the surface of graphite increased when the voltage increased from 3 V to 5 V. According to the pore size distribution measured from the SEM images, the average pore diameter is smaller for the sample prepared under an applied DC voltage of 4 V rather than 3 V, because of the sample's more compact and tightly interpenetrating network. However, the hydrogel had a greater average pore diameter when prepared under a DC voltage of 5 V rather than 4 V and a non-uniform pore structure with the largest standard deviation of pore size distribution. We believe these results might be caused by a rapid increase in oxygen generated from the electrolysis of water.

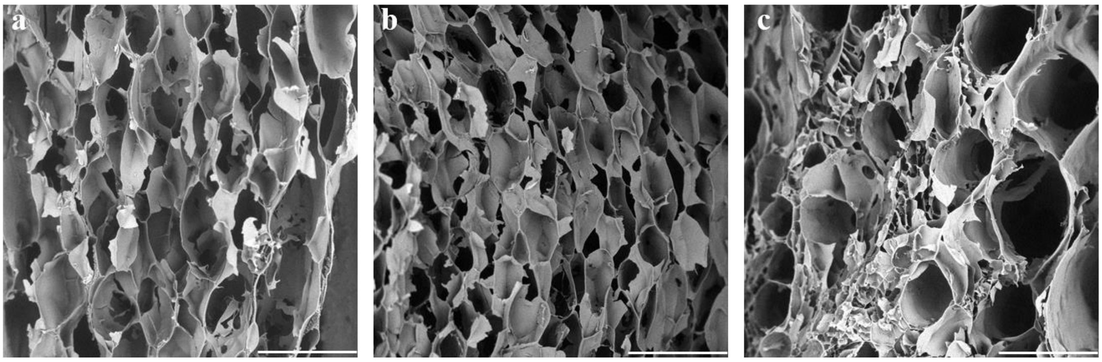

Figure S3. Microstructure of SF/CMCS hydrogels prepared from the 5% SF and 2% CMCS mixture under different voltages: (**a**) 3 V, (**b**) 4 V, and (**c**) 5 V (scale bar: 300 μm).

Table S1. Average pore size of the SF/CMCS hydrogels presented in Figure S3.

Voltage applied	Average pore diameter (μm)	
3 V	155.76 ± 40.28 μm	** (3 V vs 4 V)
4 V	98.27 ± 17.45 μm	** (4 V vs 5 V)
5 V	170.70 ± 94.19 μm	

Statistical analysis was performed using an unpaired, two-tailed t-test (* $p < 0.05$, ** $p < 0.01$, $n = 100$).

In addition, we found that the SF/CMCS hydrogel prepared under a DC voltage of 3 V demonstrated a water vapor transmission rate (WVTR) of 3008.0 ± 74.7 g·m^{-2}·day^{-1} (Figure S4), which deviated from the ideal WVTR for wound dressing (2000–2500 g·m^{-2}·day^{-1}) [3]. Therefore, a driving voltage of 4 V in DC mode was selected for SF/CMCS hydrogel preparation.

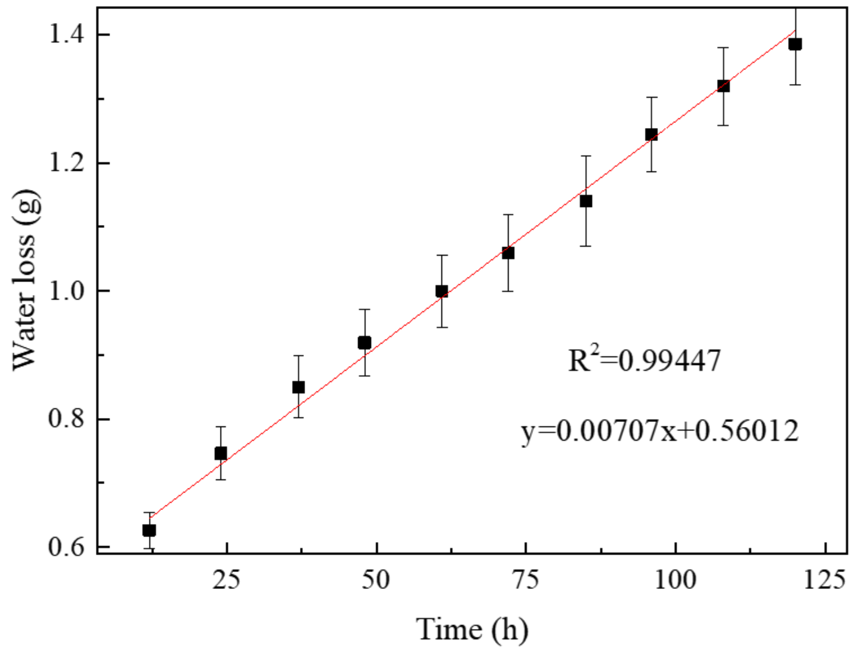

Figure S4. Water vapor transmitted across the SF/CMCS hydrogel prepared from the 5% SF and 2% CMCS mixture under a DC voltage of 3 V.

Our experiments show that 4% is the maximum achievable mass/volume ratio of the CMCS aqueous solution. When mixing 4% CMCS solution and 12% SF solution in a volume ratio of 1:1, the two-component mixture demonstrates a laminated structure, as illustrated in Figure S5. This result is due to the formation of hydrogen bonds between SF and CMCS molecules, which reduces the number of functional groups that can bind to water molecules and tends to decrease the solubility of the components. To maintain the amount of CMCS and SF at maximum, the solution containing 5% SF and 2% CMCS was used for the hydrogel preparation.

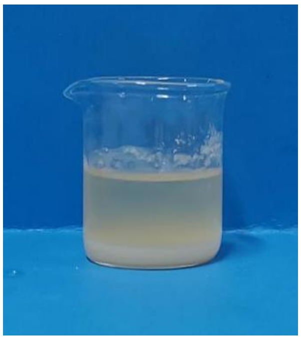

Figure S5. Aqueous mixture containing 6% SF and 2% CMCS exhibited a laminated structure.

2. Determination of silver ion leakage from Ag/AgCl reference electrode

The electrodeposition was carried out in the absence of both SF and CMCS under a voltage in DC mode of 4 V over 10 minutes by chronoamperometry. The solution was then subjected

to analysis of element silver by inductively coupled plasma mass spectrometer (ICP-MS, 7800ce, Agilent Technologies, Inc., Palo Alto, CA, USA). There was no presence of silver in the solution detected after 10 minutes of electrodeposition, indicating no leakage or diffusion of silver ions from Ag/AgCl reference electrode in the environment of electrodeposition over time.

3. Preparation of buffer solutions with different pH values for swelling study

3.1 Preparation of amino acetic acid-hydrochloric acid buffer, pH 2.1

Dissolve 0.75 g of aminoacetic acid in 500 mL of deionized water. Add 2.4 mL of hydrochloric acid (36.0%~38.0%) and 201 mL of 1 mol/L KCl solution. Dilute to 1 L with deionized water.

3.2 Preparation of saline (phosphate-buffered), pH 7.4

Dissolve 0.05 g KCl, 0.36 g Na_2HPO_4, and 0.06 g KH_2PO_4 with 200 mL of deionized water, adjust the pH 7.4 with 1 mol/L hydrochloric acid solution, and add sufficient deionized water to prepare 250 mL of saline solution.

3.3 Boric acid-potassium chloride-sodium hydroxide buffer, pH 10.0

Prepare 25 mL of 0.2 mol/L boric acid solution in a suitable container. Add 43.9 mL of 0.1 mol/L NaOH solution and 66.12 mL of 1 mol/L KCl solution to the boric acid solution. Add deionized water until the volume is 500 mL.

3.4 Disodium hydrogen phosphate-sodium hydroxide buffer solution, pH 12.1

Combine 26.9 mL of 0.1 mol/L NaOH solution, 50.0 mL of 0.05 mol/L Na_2HPO_4 solution, and 87.82 mL of 1 mol/L KCl solution. Dilute to 500 mL with deionized water.

All the buffer solutions prepared were sterilized by autoclaving at 121 °C for 20 min prior to use.

4. Representative results for antibacterial test

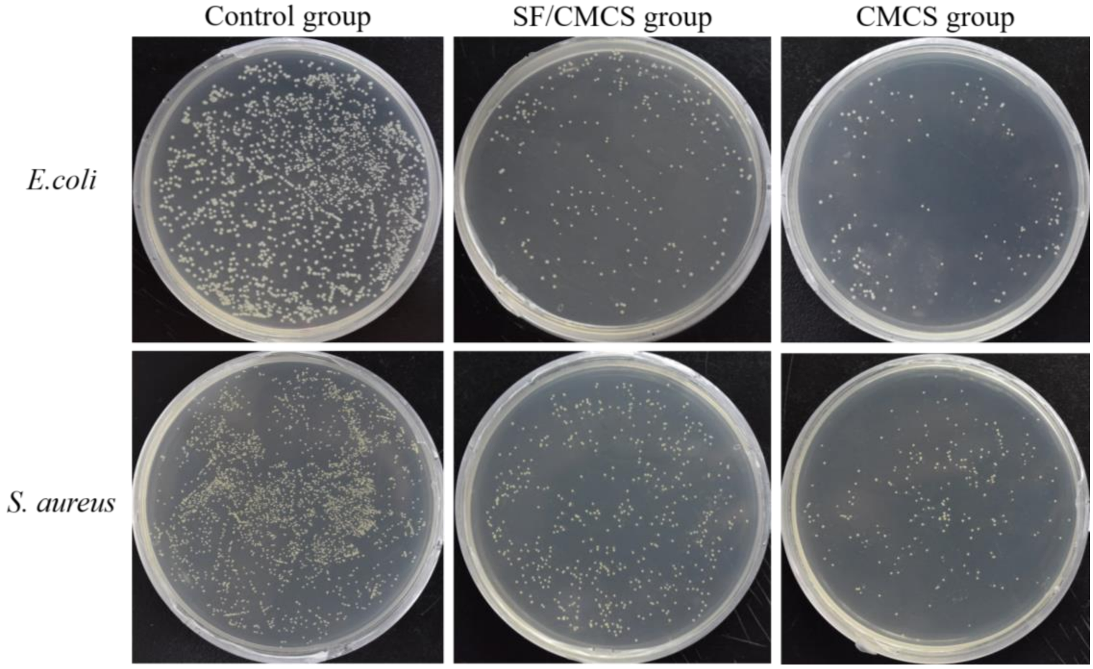

Figure S6. Representative results of the antibacterial activity of each group toward *E.coli* and *S. aureus*.

5. Confirmation of antibacterial property of the hydrogel

Following the antimicrobial assays, the hydrogels immersed in *E. coli* and *S. aureus* cell cultures were removed and rinsed with sterilized LB solution thoroughly. Subsequently, the samples were immersed in 20 mL of LB broth and cultured at 37 ℃ with a rotational speed of 100 rpm. 200 μL of LB culture were then withdrawn, and the OD_{600} values were recorded using a microplate reader (Bio-rad, Hercules, San Francisco, CA, USA) at 1 hour interval for 7 hours.

As depicted in Figure S7, there was almost no bacterial growth for both *E. coli* and *S. aureus* groups, indicating that no viable bacteria were trapped within the hydrogel, and providing further evidence for its antimicrobial activity thereafter.

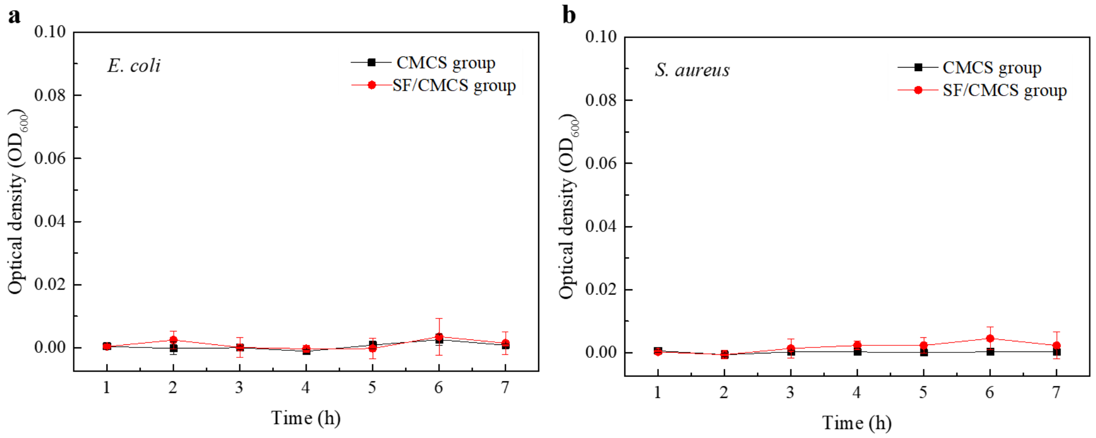

Figure S7. Growth curves of *E. coli* (**a**) and *S. aureus* (**b**) for the SF/CMCS hydrogel and the CMCS hydrogel taken out from the LB solution following the antimicrobial assays for 7 hours.

6. Morphologies of HEK 293 cells in CCK-8 assay

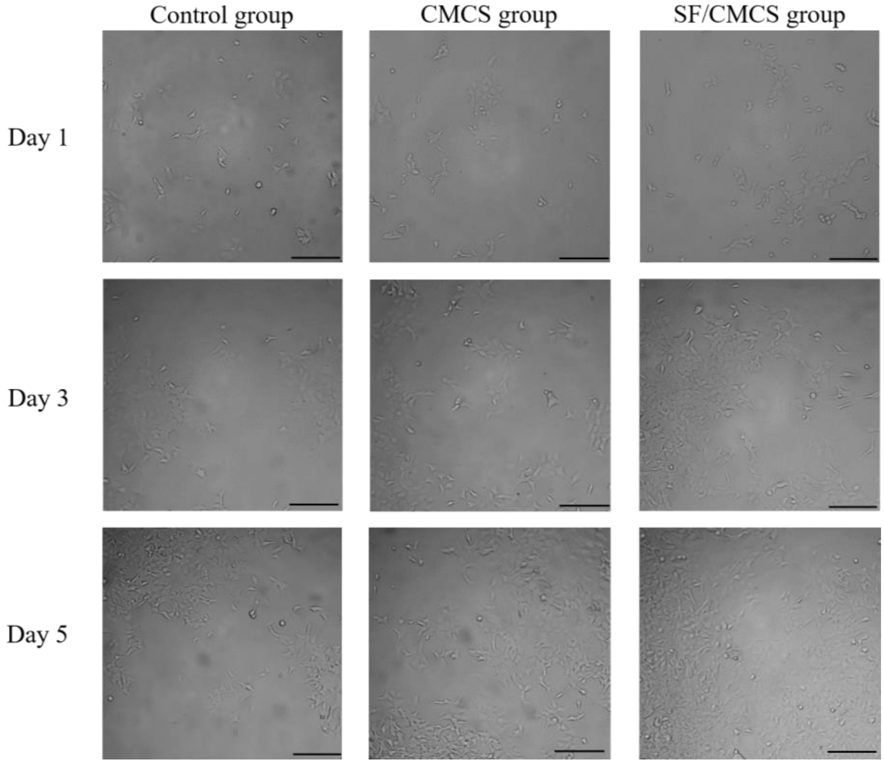

Figure S8. Morphologies of the HEK-293 cells of each group in the CCK-8 assay on day 1, day 3, and day 5 (scale bar: 200 μm).

7. Degree of wound closure

Table S2. Degree of wound closure of each group on day 3, day 7, and day 11.

Different groups	Day3	Day7	Day11
Sterile gauze group	6.29 ± 0.98%	52.82 ± 4.86%	84.05 ± 0.52%
CMCS group	14.20 ± 6.05%	60.77 ± 2.34%	90.54 ± 0.64%
CMCS/SF group	57.09 ± 4.10%	83.03 ± 0.66%	91.87 ± 0.61%

Statistical analysis was performed using an unpaired, two-tailed t-test (* $p < 0.05$, ** $p < 0.01$, $n = 3$).

8. Standard calibration curves of SF under different conditions

The concentration of SF protein in various media was measured via the Bradford assay [4].

To estimate the percentage content of SF in SF/CMCS hydrogel, the standard calibration curves of SF in the matrix of 2% CMCS solution before and after the electrodeposition of CMCS hydrogel, with SF concentrations of 0, 0.125, 0.25, 0.5, 0.75, 1.0, 1.5, 5, 7.5, and 10 mg/mL, were created (Figure S9 a,b).

To determine the SF released from SF/CMCS hydrogel in water, SF dissolved in water with concentrations of 0, 0.125, 0.25, 0.5, 0.75, 1.0, 1.5, 5, 7.5, and 10 mg/mL were prepared to create a standard calibration curve, which is shown in Figure S9c.
The correlation coefficients of all standard curves are greater than 0.99 [5, 6].

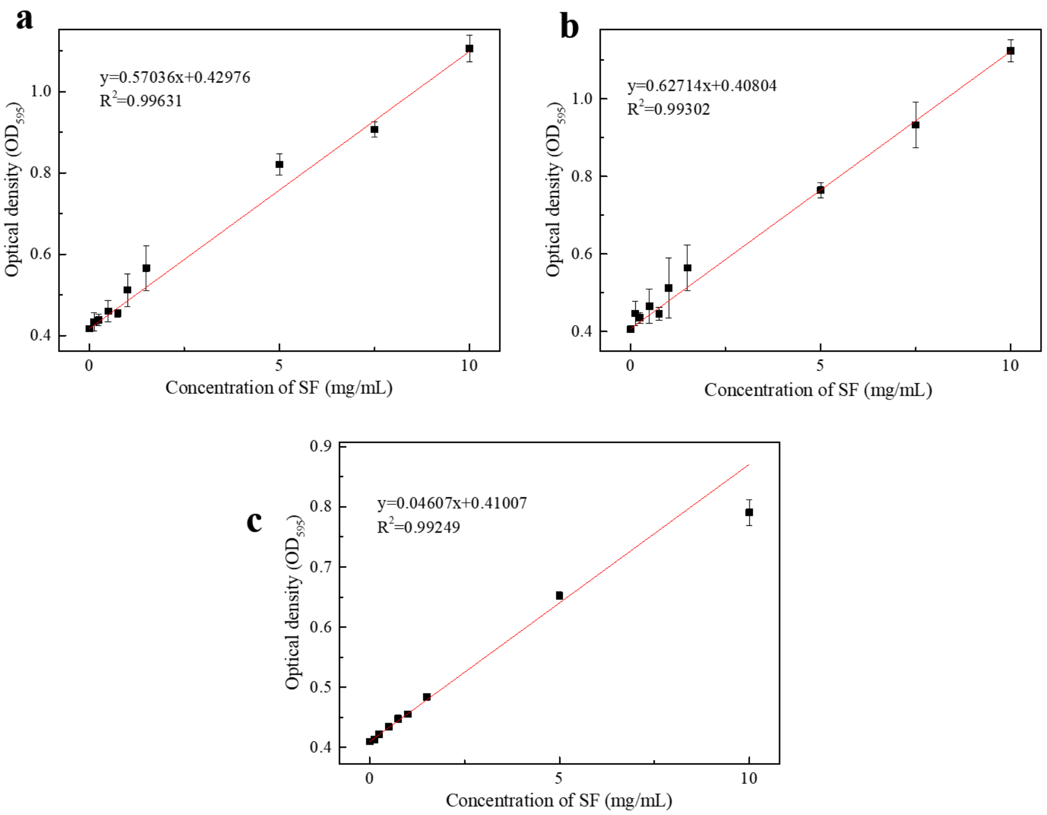

Figure S9. Calibration curves of the SF concentration in SF/CMCS solution (**a**), in the remaining solution after hydrogel preparation (**b**), and in SF solution (**c**).

References

1. Bressner, J. E.; Marelli, B.; Qin, G.; Klinker, L. E.; Zhang, Y.; Kaplan, D. L.; Omenetto, F. G., Rapid fabrication of silk films with controlled architectures via electrogelation. *J. Mater. Chem. B* **2014,** *2*, 4983-4987. https://doi.org/10.1039/c4tb00833b
2. Zahn, M., Electro-optic field and space-charge mapping measurements in high-voltage stressed dielectrics. *Phys Techn* **1985,** *16*, 288-295. https://doi.org/10.1088/0305-4624/16/6/I03
3. Queen, D.; Gaylor, J. D. S.; Evans, J. H.; Courtney, J. M.; Reid, W. H., The preclinical evaluation of the water vapour transmission rate through burn wound dressings. *Biomaterials* **1987,** *8*, 367-371. https://doi.org/10.1016/0142-9612(87)90007-X
4. Aneesia, V.; Elakkiya, V.; Ponjanani, S.; Gopinathan, J.; Amitava, B., Impact of silk fibroin-based scaffold structures on human osteoblast MG63 cell attachment and proliferation. *Int. J. Nanomed* **2015,** *10*, 43-51. https://doi.org/10.2147/IJN.S82209
5. Farías, T.; Ménorval, L. C. d.; Zajac, J.; Rivera, A., Adsolubilization of drugs onto natural clinoptilolite modified by adsorption of cationic surfactants. *Colloid. Surface. B* **2010,** *76*, 421-426. https://doi.org/10.1016/j.colsurfb.2009.11.018
6. Alexopoulou, E.; Stripeli, F.; Baras, P.; Seimenis, I.; Kelekis, N. L., R2 relaxometry with MRI for the quantification of tissue iron overload in beta-thalassemic patients. *J. Magn. Reson. Imaging* **2010,** *23*, 163-170. https://doi.org/10.1002/jmri.20489
